# Piperidine-1-carboxamidinium ethyl carbonate

**DOI:** 10.1107/S1600536812045497

**Published:** 2012-11-10

**Authors:** Ioannis Tiritiris

**Affiliations:** a Fakultät Chemie/Organische Chemie, Hochschule Aalen, Beethovenstrasse 1, D-73430 Aalen, Germany

## Abstract

In the title salt, C_6_H_14_N_3_^+^·C_3_H_5_O_3_^−^, the C—N bond lengths in the central CN_3_ unit of the carboxamidinium cation are 1.3262 (18), 1.3359 (18) and 1.3498 (18) Å, indicating partial double-bond character. The central C atom is bonded to the three N atoms in a nearly ideal trigonal–planar geometry and the positive charge is delocalized in the CN_3_ plane. The piperidine ring is in a chair conformation. The C—O bond lengths in the ethyl carbonate anion are characteristic for a delocalized double bond and a typical single bond. In the crystal, N—H⋯O hydrogen bonds between cations and anions generate a two-dimensional network in the direction of the *ab* plane, whereas adjacent ion pairs form chains running along the *b* axis.

## Related literature

For the synthesis and crystal structures of guanidinium hydrogencarbonates, see: Tiritiris *et al.* (2011 ▶). For the crystal structure of piperidine-1-carboximidamide, see: Tiritiris (2012 ▶), and for the crystal structure of sodium methyl carbonate, see: Kunert *et al.* (1998 ▶).
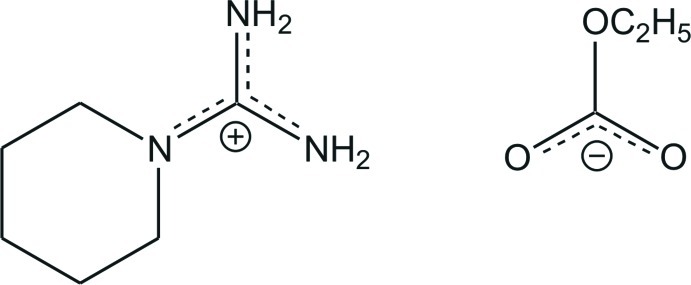


## Experimental

### 

#### Crystal data

C_6_H_14_N_3_^+^·C_3_H_5_O_3_^−^
*M* *_r_* = 217.27
Monoclinic, 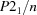

*a* = 11.8320 (6) Å
*b* = 7.2407 (4) Å
*c* = 13.3755 (9) Å
β = 105.292 (3)°
*V* = 1105.33 (11) Å^3^
*Z* = 4
Mo *K*α radiation
μ = 0.10 mm^−1^
*T* = 100 K
0.25 × 0.20 × 0.05 mm

#### Data collection

Bruker–Nonius KappaCCD diffractometer
4452 measured reflections
2638 independent reflections
1982 reflections with *I* > 2σ(*I*)
*R*_int_ = 0.047

#### Refinement

*R*[*F* ^2^ > 2σ(*F* ^2^)] = 0.042
*wR*(*F* ^2^) = 0.106
*S* = 1.02
2638 reflections
153 parameters
H atoms treated by a mixture of independent and constrained refinement
Δρ_max_ = 0.28 e Å^−3^
Δρ_min_ = −0.23 e Å^−3^

### 

Data collection: *COLLECT* (Hooft, 2004 ▶); cell refinement: *SCALEPACK* (Otwinowski & Minor, 1997 ▶); data reduction: *SCALEPACK*; program(s) used to solve structure: *SHELXS97* (Sheldrick, 2008 ▶); program(s) used to refine structure: *SHELXL97* (Sheldrick, 2008 ▶); molecular graphics: *DIAMOND* (Brandenburg & Putz, 2005 ▶); software used to prepare material for publication: *SHELXL97*.

## Supplementary Material

Crystal structure: contains datablock(s) I, global. DOI: 10.1107/S1600536812045497/kp2439sup1.cif

Structure factors: contains datablock(s) I. DOI: 10.1107/S1600536812045497/kp2439Isup2.hkl

Additional supplementary materials:  crystallographic information; 3D view; checkCIF report

## Figures and Tables

**Table 1:** Hydrogen-bond geometry (Å, °)

*D*—H⋯*A*	*D*—H	H⋯*A*	*D*⋯*A*	*D*—H⋯*A*
N1—H11⋯O2^i^	0.88 (2)	1.99 (2)	2.812 (1)	155 (1)
N1—H12⋯O2^ii^	0.88 (2)	1.88 (2)	2.747 (1)	173 (1)
N2—H21⋯O1^ii^	0.84 (2)	2.19 (2)	3.033 (1)	175 (1)
N2—H22⋯O1^iii^	0.87 (2)	2.06 (2)	2.923 (1)	170 (1)

Symmetry codes: (i) 
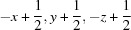
; (ii) 
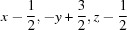
; (iii) 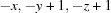.

**Fig. 1.**
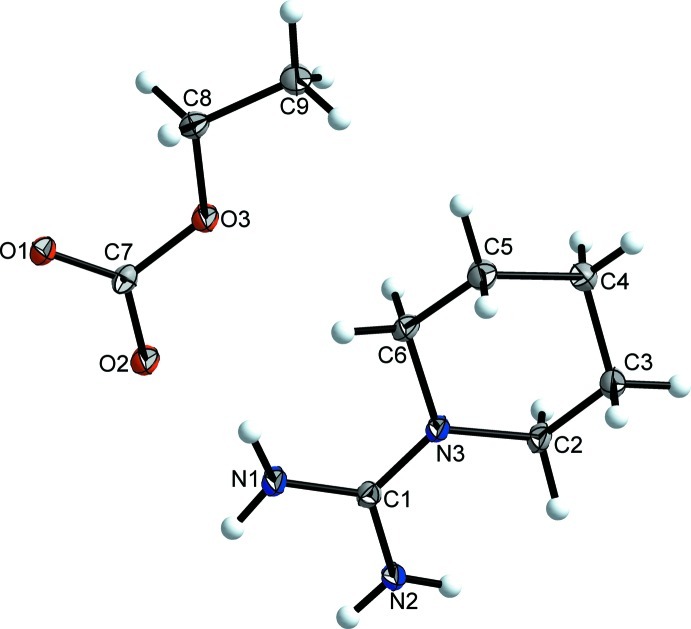
The structure of the title compound with displacement ellipsoids at the 50% probability level.

**Fig. 2.**
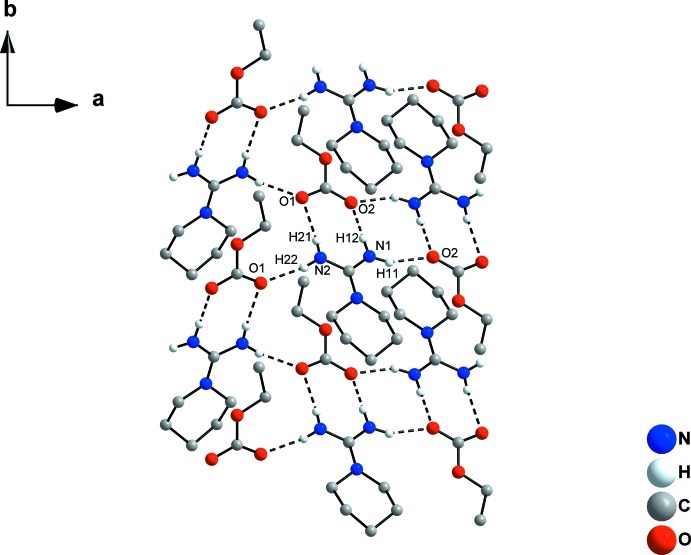
N–H···O hydrogen bonds generating a two-dimensional network, ab-view. The hydrogen bonds are indicated by dashed lines.

**Fig. 3.**
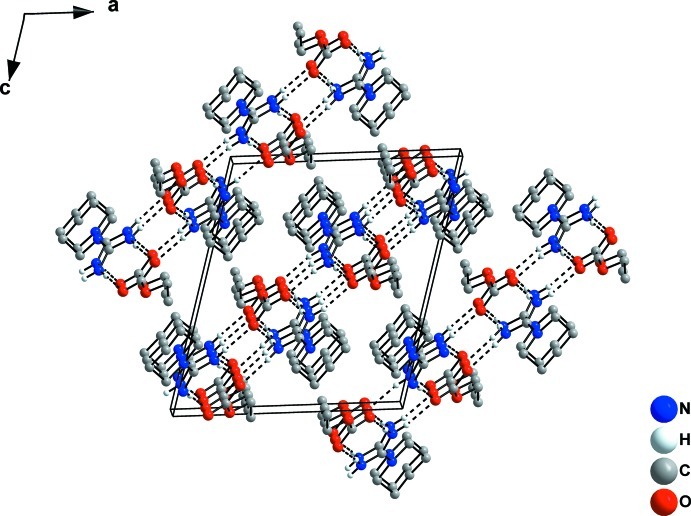
N–H···O hydrogen bond arrangement forming chains running along the b axis. The hydrogen bonds are indicated by dashed lines.

## supplementary crystallographic information

### Comment

By reacting guanidines with CO_2_ in undried aprotic solvents, the corresponding guanidinium hydrogen carbonate salts are formed exclusively (Tiritiris *et al.*, 2011). To investigate the reaction of carboxamidines with CO_2_, we used both aprotic and protic solvents. Due to the water content in the common aprotic solvents, the hydrogen carbonate salts were formed too. Most of them are sparingly soluble and could therefore not be obtained in crystalline form. By using ethanol as a solvent for the reaction, the crystalline title compound emerged. According to the structure analysis, the C1–N1 bond in the title compound is 1.3262 (18) Å, C1–N2 = 1.3359 (18) Å and C1–N3 = 1.3498 (18) Å, showing partial double-bond character (Fig. 1). The N–C1–N angles are: 117.59 (13)° (N1–C1–N2), 121.04 (12)° (N1–C1–N3) and 121.36 (12)° (N2–C1–N3), which indicate a nearly ideal trigonal-planar surrounding of the carbon centre by the nitrogen atoms. The positive charge is completely delocalized on the CN_3_ plane. The structural parameters of the piperidine ring in the here presented title compound agree very well with the data obtained from the X-ray analysis of the starting compound piperidine-1-carboximidamide (Tiritiris, 2012). The piperidine ring adopt a chair conformation. In the ethyl carbonate ion the C7–O1 and C7–O2 bond lengths indicate an evenly distributed double bond character (C7–O1, 1.2485 (16) Å; C7–O2, 1.2509 (17) Å) and a typical single bond (C7–O3, 1.3706 (18) Å). The data fit with the C–O bond lengths of the anion in sodium methyl carbonate (Kunert *et al.*, 1998). In the crystal structure, strong N—H···O hydrogen bonds between hydrogen atoms of carboxamidinium ions and oxygen atoms of neighboring ethyl carbonate ions are observed, generating an infinite two-dimensional network [*d*(H···O) = 1.88 (2)–2.19 (2) Å] (Tab. 1) with base vectors [1 0 - 1] and [0 1 0] (Fig. 2). Furthermore, the hydrogen bonds are arranged in a way, that adjacent ion pairs are forming chains running along the *b* axis (Fig. 3).

### Experimental

The title compound was prepared by bubbling excess CO_2_ gas into an ethanolic solution of 2.04 g (16 mmol) piperidine-1-carboximidamide (Tiritiris, 2012). The resulting colourless precipitate was recrystallized from a small amount of ethanol and single crystals suitable for X-ray analysis were obtained. Yield: 3.25 g (93.3%). ^1^H NMR (500 MHz, D_2_O/DSS): δ = 1.17–1.20 [t, 3 H, –CH_3_], 1.61–1.70 [m, 6 H, –CH_2_], 3.40–3.43 [m, 4 H, –CH_2_], 3.64–3.68 [q, 2 H, –CH_2_]. Because of the H/D exchange, the hydrogen atoms of the –NH_2_ groups were not observed. ^13^C NMR (125 MHz, D_2_O/DSS): δ = 16.8 (–CH_3_), 23.1 (–CH_2_), 24.7 (–CH_2_), 46.7 (–CH_2_), 57.4 (–CH_2_), 155.5 (N_3_C^+^), 160.3 (C═O).

### Refinement

The N-bound H atoms were located in a difference Fourier map and were refined freely [N—H = 0.84 (2)–0.88 (2) Å]. The hydrogen atoms of the methyl group were allowed to rotate with a fixed angle around the C–C bond to best fit the experimental electron density, with *U*(H) set to 1.5 *U*_eq_(C) and d(C—H) = 0.98 Å. The H atoms of the methylene groups were placed in calculated positions with d(C—H) = 0.99 Å. They were included in the refinement in the riding model approximation, with *U*(H) set to 1.2 *U*_eq_(C).

### Crystal data

**Table:**

C_6_H_14_N_3_^+^·C_3_H_5_O_3_^−^	*F*(000) = 472
*M**_r_* = 217.27	*D*_x_ = 1.306 Mg m^−^^3^
Monoclinic, *P*2_1_/*n*	Melting point: 397 K
Hall symbol: -P 2yn	Mo *K*α radiation, λ = 0.71073 Å
*a* = 11.8320 (6) Å	Cell parameters from 2732 reflections
*b* = 7.2407 (4) Å	θ = 0.4–27.9°
*c* = 13.3755 (9) Å	µ = 0.10 mm^−^^1^
β = 105.292 (3)°	*T* = 100 K
*V* = 1105.33 (11) Å^3^	Plate, colourless
*Z* = 4	0.25 × 0.20 × 0.05 mm

### Data collection

**Table:**

Bruker–Nonius KappaCCD diffractometer	1982 reflections with *I* > 2σ(*I*)
Radiation source: sealed tube	*R*_int_ = 0.047
Graphite monochromator	θ_max_ = 27.9°, θ_min_ = 2.1°
φ scans, and ω scans	*h* = −15→15
4452 measured reflections	*k* = −9→8
2638 independent reflections	*l* = −17→17

### Refinement

**Table:**

Refinement on *F*^2^	Primary atom site location: structure-invariant direct methods
Least-squares matrix: full	Secondary atom site location: difference Fourier map
*R*[*F*^2^ > 2σ(*F*^2^)] = 0.042	Hydrogen site location: difference Fourier map
*wR*(*F*^2^) = 0.106	H atoms treated by a mixture of independent and constrained refinement
*S* = 1.02	*w* = 1/[σ^2^(*F*_o_^2^) + (0.0412*P*)^2^ + 0.424*P*] where *P* = (*F*_o_^2^ + 2*F*_c_^2^)/3
2638 reflections	(Δ/σ)_max_ < 0.001
153 parameters	Δρ_max_ = 0.28 e Å^−^^3^
0 restraints	Δρ_min_ = −0.23 e Å^−^^3^

### Special details

**Table:**

Geometry. All e.s.d.'s (except the e.s.d. in the dihedral angle between two l.s. planes) are estimated using the full covariance matrix. The cell e.s.d.'s are taken into account individually in the estimation of e.s.d.'s in distances, angles and torsion angles; correlations between e.s.d.'s in cell parameters are only used when they are defined by crystal symmetry. An approximate (isotropic) treatment of cell e.s.d.'s is used for estimating e.s.d.'s involving l.s. planes.
Refinement. Refinement of *F*^2^ against ALL reflections. The weighted *R*-factor *wR* and goodness of fit *S* are based on *F*^2^, conventional *R*-factors *R* are based on *F*, with *F* set to zero for negative *F*^2^. The threshold expression of *F*^2^ > σ(*F*^2^) is used only for calculating *R*-factors(gt) *etc*. and is not relevant to the choice of reflections for refinement. *R*-factors based on *F*^2^ are statistically about twice as large as those based on *F*, and *R*- factors based on ALL data will be even larger.

### Fractional atomic coordinates and isotropic or equivalent isotropic displacement parameters (Å^2^)

**Table:**

	*x*	*y*	*z*	*U*_iso_*/*U*_eq_	
N1	−0.01610 (11)	0.84504 (19)	0.10282 (10)	0.0182 (3)	
H11	0.0373 (17)	0.810 (3)	0.0712 (16)	0.030 (5)*	
H12	−0.0527 (16)	0.949 (3)	0.0829 (15)	0.029 (5)*	
N2	−0.11973 (10)	0.83590 (18)	0.22402 (10)	0.0162 (3)	
H21	−0.1508 (14)	0.936 (3)	0.1994 (14)	0.019 (4)*	
H22	−0.1552 (15)	0.779 (3)	0.2649 (15)	0.023 (5)*	
N3	0.01511 (10)	0.60093 (16)	0.22039 (9)	0.0140 (2)	
C1	−0.04041 (11)	0.75874 (19)	0.18211 (11)	0.0138 (3)	
C2	−0.02711 (12)	0.4939 (2)	0.29709 (11)	0.0163 (3)	
H2A	−0.0969	0.4214	0.2610	0.020*	
H2B	−0.0508	0.5798	0.3455	0.020*	
C3	0.06700 (13)	0.3636 (2)	0.35824 (11)	0.0192 (3)	
H3A	0.1322	0.4369	0.4019	0.023*	
H3B	0.0338	0.2866	0.4046	0.023*	
C4	0.11384 (13)	0.2397 (2)	0.28736 (12)	0.0191 (3)	
H4A	0.0498	0.1632	0.2446	0.023*	
H4B	0.1748	0.1564	0.3288	0.023*	
C5	0.16564 (13)	0.3613 (2)	0.21824 (12)	0.0219 (3)	
H5A	0.2320	0.4326	0.2617	0.026*	
H5B	0.1963	0.2825	0.1709	0.026*	
C6	0.07529 (12)	0.4939 (2)	0.15493 (11)	0.0188 (3)	
H6A	0.1144	0.5805	0.1175	0.023*	
H6B	0.0163	0.4232	0.1027	0.023*	
O1	0.25289 (8)	0.31135 (14)	0.63820 (8)	0.0173 (2)	
O2	0.36182 (9)	0.34098 (15)	0.52507 (8)	0.0210 (2)	
O3	0.26553 (8)	0.08408 (14)	0.52492 (8)	0.0175 (2)	
C7	0.29380 (11)	0.2563 (2)	0.56646 (11)	0.0144 (3)	
C8	0.18221 (12)	−0.0237 (2)	0.56032 (11)	0.0169 (3)	
H8A	0.1079	0.0451	0.5511	0.020*	
H8B	0.2132	−0.0548	0.6346	0.020*	
C9	0.16276 (14)	−0.1971 (2)	0.49492 (12)	0.0232 (3)	
H9A	0.1235	−0.1652	0.4229	0.035*	
H9B	0.1137	−0.2832	0.5215	0.035*	
H9C	0.2384	−0.2551	0.4980	0.035*	

### Atomic displacement parameters (Å^2^)

**Table:**

	*U*^11^	*U*^22^	*U*^33^	*U*^12^	*U*^13^	*U*^23^
N1	0.0210 (6)	0.0191 (6)	0.0183 (6)	0.0056 (5)	0.0120 (5)	0.0059 (5)
N2	0.0193 (6)	0.0147 (6)	0.0175 (6)	0.0039 (5)	0.0100 (5)	0.0048 (5)
N3	0.0151 (5)	0.0161 (6)	0.0124 (5)	0.0021 (4)	0.0064 (4)	0.0022 (5)
C1	0.0155 (6)	0.0145 (6)	0.0115 (6)	−0.0020 (5)	0.0037 (5)	−0.0012 (5)
C2	0.0177 (6)	0.0194 (7)	0.0141 (7)	0.0018 (6)	0.0086 (5)	0.0035 (6)
C3	0.0228 (7)	0.0207 (7)	0.0154 (7)	0.0043 (6)	0.0072 (6)	0.0048 (6)
C4	0.0243 (7)	0.0145 (7)	0.0207 (7)	0.0026 (6)	0.0098 (6)	0.0027 (6)
C5	0.0232 (7)	0.0229 (8)	0.0240 (8)	0.0081 (6)	0.0137 (6)	0.0062 (6)
C6	0.0229 (7)	0.0206 (7)	0.0168 (7)	0.0060 (6)	0.0122 (6)	0.0028 (6)
O1	0.0201 (5)	0.0178 (5)	0.0171 (5)	−0.0018 (4)	0.0104 (4)	−0.0027 (4)
O2	0.0244 (5)	0.0221 (6)	0.0209 (5)	−0.0078 (4)	0.0139 (4)	−0.0052 (4)
O3	0.0188 (5)	0.0184 (5)	0.0181 (5)	−0.0050 (4)	0.0099 (4)	−0.0041 (4)
C7	0.0123 (6)	0.0181 (7)	0.0131 (7)	0.0006 (5)	0.0037 (5)	−0.0003 (5)
C8	0.0169 (6)	0.0186 (7)	0.0174 (7)	−0.0021 (5)	0.0081 (6)	−0.0004 (6)
C9	0.0250 (7)	0.0238 (8)	0.0241 (8)	−0.0083 (6)	0.0121 (6)	−0.0052 (7)

### Geometric parameters (Å, º)

**Table:**

N1—C1	1.3262 (18)	C4—H4B	0.9900
N1—H11	0.88 (2)	C5—C6	1.518 (2)
N1—H12	0.88 (2)	C5—H5A	0.9900
N2—C1	1.3359 (18)	C5—H5B	0.9900
N2—H21	0.84 (2)	C6—H6A	0.9900
N2—H22	0.87 (2)	C6—H6B	0.9900
N3—C1	1.3498 (18)	O1—C7	1.2485 (16)
N3—C2	1.4742 (17)	O2—C7	1.2509 (17)
N3—C6	1.4842 (17)	O3—C7	1.3706 (18)
C2—C3	1.5227 (19)	O3—C8	1.4323 (16)
C2—H2A	0.9900	C8—C9	1.512 (2)
C2—H2B	0.9900	C8—H8A	0.9900
C3—C4	1.512 (2)	C8—H8B	0.9900
C3—H3A	0.9900	C9—H9A	0.9800
C3—H3B	0.9900	C9—H9B	0.9800
C4—C5	1.518 (2)	C9—H9C	0.9800
C4—H4A	0.9900		
			
C1—N1—H11	125.4 (13)	H4A—C4—H4B	108.4
C1—N1—H12	117.1 (12)	C6—C5—C4	111.62 (12)
H11—N1—H12	117.4 (18)	C6—C5—H5A	109.3
C1—N2—H21	118.6 (11)	C4—C5—H5A	109.3
C1—N2—H22	125.0 (12)	C6—C5—H5B	109.3
H21—N2—H22	114.7 (16)	C4—C5—H5B	109.3
C1—N3—C2	119.25 (11)	H5A—C5—H5B	108.0
C1—N3—C6	118.65 (11)	N3—C6—C5	112.21 (11)
C2—N3—C6	116.07 (11)	N3—C6—H6A	109.2
N1—C1—N2	117.59 (13)	C5—C6—H6A	109.2
N1—C1—N3	121.04 (12)	N3—C6—H6B	109.2
N2—C1—N3	121.36 (12)	C5—C6—H6B	109.2
N3—C2—C3	111.43 (10)	H6A—C6—H6B	107.9
N3—C2—H2A	109.3	C7—O3—C8	118.52 (10)
C3—C2—H2A	109.3	O1—C7—O2	127.52 (13)
N3—C2—H2B	109.3	O1—C7—O3	119.95 (12)
C3—C2—H2B	109.3	O2—C7—O3	112.53 (12)
H2A—C2—H2B	108.0	O3—C8—C9	105.90 (11)
C4—C3—C2	111.57 (12)	O3—C8—H8A	110.6
C4—C3—H3A	109.3	C9—C8—H8A	110.6
C2—C3—H3A	109.3	O3—C8—H8B	110.6
C4—C3—H3B	109.3	C9—C8—H8B	110.6
C2—C3—H3B	109.3	H8A—C8—H8B	108.7
H3A—C3—H3B	108.0	C8—C9—H9A	109.5
C3—C4—C5	108.14 (12)	C8—C9—H9B	109.5
C3—C4—H4A	110.1	H9A—C9—H9B	109.5
C5—C4—H4A	110.1	C8—C9—H9C	109.5
C3—C4—H4B	110.1	H9A—C9—H9C	109.5
C5—C4—H4B	110.1	H9B—C9—H9C	109.5
			
C2—N3—C1—N1	−170.39 (13)	C3—C4—C5—C6	−58.57 (17)
C6—N3—C1—N1	−18.76 (19)	C1—N3—C6—C5	160.64 (12)
C2—N3—C1—N2	11.15 (19)	C2—N3—C6—C5	−46.85 (16)
C6—N3—C1—N2	162.78 (13)	C4—C5—C6—N3	52.04 (17)
C1—N3—C2—C3	−160.06 (12)	C8—O3—C7—O1	4.51 (19)
C6—N3—C2—C3	47.59 (16)	C8—O3—C7—O2	−175.98 (12)
N3—C2—C3—C4	−54.20 (16)	C7—O3—C8—C9	175.66 (12)
C2—C3—C4—C5	59.74 (16)		

### Hydrogen-bond geometry (Å, º)

**Table:**

*D*—H···*A*	*D*—H	H···*A*	*D*···*A*	*D*—H···*A*
N1—H11···O2^i^	0.88 (2)	1.99 (2)	2.812 (1)	155 (1)
N1—H12···O2^ii^	0.88 (2)	1.88 (2)	2.747 (1)	173 (1)
N2—H21···O1^ii^	0.84 (2)	2.19 (2)	3.033 (1)	175 (1)
N2—H22···O1^iii^	0.87 (2)	2.06 (2)	2.923 (1)	170 (1)

Symmetry codes: (i) −*x*+1/2, *y*+1/2, −*z*+1/2; (ii) *x*−1/2, −*y*+3/2, *z*−1/2; (iii) −*x*, −*y*+1, −*z*+1.
